# Natural and molecular history of prolactinoma: insights from a *Prlr* ^–/–^ mouse model

**DOI:** 10.18632/oncotarget.23713

**Published:** 2017-12-27

**Authors:** Valérie Bernard, Chiara Villa, Aurélie Auguste, Sophie Lamothe, Anne Guillou, Agnès Martin, Sandrine Caburet, Jacques Young, Reiner A. Veitia, Nadine Binart

**Affiliations:** ^1^ Unité INSERM 1185, Faculté de Médecine Paris Sud, Université Paris-Saclay, le Kremlin-Bicêtre, France; ^2^ Service d’Anatomie et Cytologie Pathologiques, Hôpital Foch, Suresnes, France; ^3^ Institut Cochin, Unité INSERM 1016, CNRS UMR 8104, Université Paris Diderot, Paris, France; ^4^ Unité INSERM 981, Institut Gustave Roussy, Université Paris-Saclay, Villejuif, France; ^5^ Unité INSERM 1191, CNRS, Institut de Génomique Fonctionnelle, Montpellier, France; ^6^ Institut Jacques Monod, Université Paris Diderot, Paris, France; ^7^ APHP, Hôpital de Bicêtre, Service d’Endocrinologie et des Maladies de la Reproduction, le Kremlin-Bicêtre, France

**Keywords:** prolactin receptor, prolactinoma, pituitary adenoma, transcriptomics, mouse model

## Abstract

Lactotroph adenoma, also called prolactinoma, is the most common pituitary tumor but little is known about its pathogenesis. Mouse models of prolactinoma can be useful to better understand molecular mechanisms involved in abnormal lactotroph cell proliferation and secretion. We have previously developed a prolactin receptor deficient (*Prlr*^*–/–*^) mouse, which develops prolactinoma. The present study aims to explore the natural history of prolactinoma formation in *Prlr*^*–/–*^ mice, using hormonal, radiological, histological and molecular analyses to uncover mechanisms involved in lactotroph adenoma development. *Prlr*^*–/–*^ females develop large secreting prolactinomas from 12 months of age, with a penetrance of 100%, mimicking human aggressive densely granulated macroprolactinoma, which is a highly secreting subtype. Mean blood PRL measurements reach 14 902 ng/mL at 24 months in *Prlr*^*–/–*^ females while PRL levels were below 15 ng/mL in control mice (*p* < 0.01). By comparing pituitary microarray data of *Prlr*^*–/–*^ mice and an estrogen-induced prolactinoma model in ACI rats, we pinpointed 218 concordantly differentially expressed (DE) genes involved in cell cycle, mitosis, cell adhesion molecules, dopaminergic synapse and estrogen signaling. Pathway/gene-set enrichment analyses suggest that the transcriptomic dysregulation in both models of prolactinoma might be mediated by a limited set of transcription factors (i.e., STAT5, STAT3, AhR, ESR1, BRD4, CEBPD, YAP, FOXO1) and kinases (i.e., JAK2, AKT1, BRAF, BMPR1A, CDK8, HUNK, ALK, FGFR1, ILK). Our experimental results and their bioinformatic analysis provide insights into early genomic changes in murine models of the most frequent human pituitary tumor.

## INTRODUCTION

Prolactin (PRL), the hormone of lactation, is synthesized and secreted by lactotroph cells of the anterior pituitary gland. Human lactotroph adenoma, also called prolactinoma, is the most common pituitary tumor, with a prevalence of about 50 per 100 000 [1–3]. The excess of PRL secretion by the tumor can result in hypogonadism, infertility and galactorrhea, whereas tumor growth can lead to compressive mass effects resulting in headache and visual defects [4]. The molecular action of PRL is exerted via a transmembrane PRL receptor (PRLR), which is a member of the haematopoietic cytokine receptor superfamily and is ubiquitously expressed including on lactotroph cells. Physiologically, PRL synthesis and secretion is under the control of multiple stimulatory and inhibitory factors. Dopamine, which is secreted by tubero-infundibular hypothalamic (TIDA) neurons, is the primary inhibitory regulator of this process [5]. Its inhibitory tone is exerted via D2 dopamine receptors located on the surface of lactotroph cells. PRL itself exerts a negative feedback effect on its own secretion. It has been demonstrated in rodents that PRL stimulates hypothalamic dopamine synthesis [6] and turnover [7, 8] and promotes dopamine secretion into the pituitary portal blood [9]. Such mechanism has been suggested to exist in humans [10].

Cabergoline, a D2-selective and potent agonist drug, has been demonstrated to be an effective treatment for prolactinomas [4]. However, about 10% of prolactinomas are resistant to this therapy, a phenomenon that is not currently understood [11]. These data point to the need for a better understanding of the pathogenesis of prolactinoma, thus animal models of lactrotroph adenomas can be useful for this purpose.

We have previously developed a model of *Prlr*-deficient (*Prlr*^–/–^) mice [12], which exhibit hyperprolactinemia and tumors associated with an increased lactrotroph cell proliferation in both sexes, with a more severe phenotype in females [13]. A loss of negative dopaminergic growth control resulting from a lack of PRL action on the hypothalamus might be at the origin of prolactinoma in this model but a direct autocrine action of PRL on lactotroph cells is not excluded [13]. Interestingly, hyperprolactinemia has recently been observed in humans bearing an inactivating heterozygous mutation of PRLR [14], reinforcing the relevance of perturbations of PRLR signaling in this pathology, and suggesting that the feedback mechanism mentioned above is conserved among mammals.

The objective of the present study is to better understand the natural history and molecular mechanisms underlying prolactinoma development in *Prlr*^–/–^ mouse model, by classical approaches such as PRL measurements and histological analysis, but also by original approaches such as pituitary MRI imaging and transcriptomic analyses.

## RESULTS AND DISCUSSION

### Natural history of prolactinoma development in mice lacking Prlr

#### Prolactin levels

**Figure 1:**
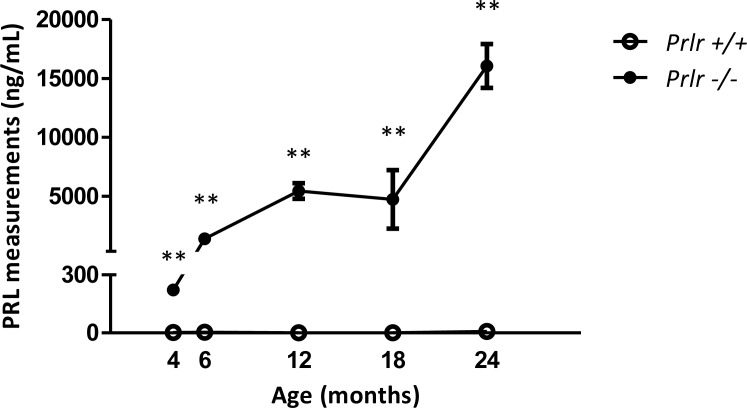
Blood PRL measurements in *Prlr*^–/–^ mice compared to *Prlr*^+/+^ mice Measurements were performed at 4, 6, 12, 18 and 24 months of age (*n =* 6 in each group). Mean ± SEM levels of PRL in mice of both genotypes **p < 0.01.

*Prlr*^–/–^ female mice displayed a marked increased PRL levels when compared to *Prlr*^+/+^ wild type counterparts, and hyperprolactinemia showed a dramatic increase with age (Figure 1). Specifically, mean blood PRL levels soared from 223 ng/mL at 4 months of age to 14 902 ng/mL at 24 months in mutant mice (*n =* 6 for each age) while PRL levels in control mice (*n =* 6 for each age) were below 15 ng/mL at all ages (*p <* 0.01).

#### Magnetic resonance imaging (MRI)

**Figure 2:**
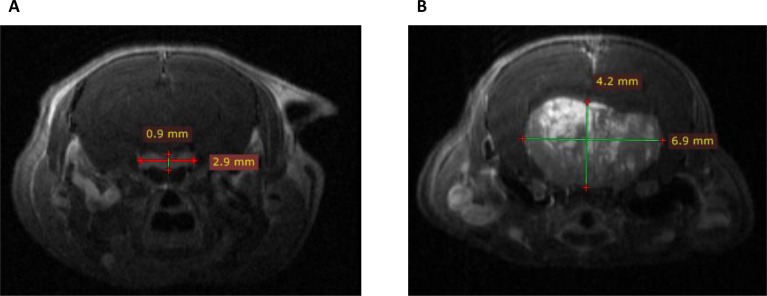
Coronal T1 weighted post-gadolinium enhanced Magnetic Resonance Imaging scans in *Prlr*^+/+^ and *Prlr*^–/–^ 18-month-old female mice (**A**) Normal pituitary gland in a *Prlr*^+/+^ mouse. Pituitary measured 2.9 × 0.9 mm, with a homogeneous signal after gadolinium injection. (**B**) Representative pituitary imaging in a *Prlr*^–/–^ mouse revealing heterogenous T1 enhanced pituitary macroadenoma of 6.9 × 4.2 mm of diameter with cerebral mass effect.

To evaluate *in vivo* lactotroph tumor development, pituitary MRI was performed in two 18-month-old *Prlr*^–/–^ female mice as compared to two *Prlr*^+*/+*^ animals (Figure 2). This imaging technology revealed the presence of large heterogeneous T1 enhanced pituitary macroadenomas reaching 6.9 mm of diameter in *Prlr*^–/–^ females, with cerebral mass effect. This heterogeneous magnetic signal after gadolinium injection suggested the existence of an important neovascularization in the tumor, a process also described in human prolactinomas [15].

#### Cabergoline treatment

Interestingly, such dramatic hyperprolactinemia observed in *Prlr*^–/–^ females was completely abolished by the administration of the dopaminergic agonist cabergoline. Two 12-month-old animals recovered normal PRL values (10 ng/mL) after daily oral administration of cabergoline (1 mg/kg/day) for one month. However, pituitary MRI performance after 1 month and 3 months of treatment did not reveal tumor size reduction, suggesting a discordance in the response of *Prlr*^–/–^ mice to dopaminergic agonist treatment. We also performed an anatomo-pathological assessment and were unable to observe neither necrosis nor changes of fibrosis, but we noticed a decreased mitotic activity compared to naïve (non treated) animals. In humans, such discordance in the response of a given patient with respect to decreased PRL levels and tumor size reduction is rare but has already been reported [16].

#### Histological analysis

**Figure 3:**
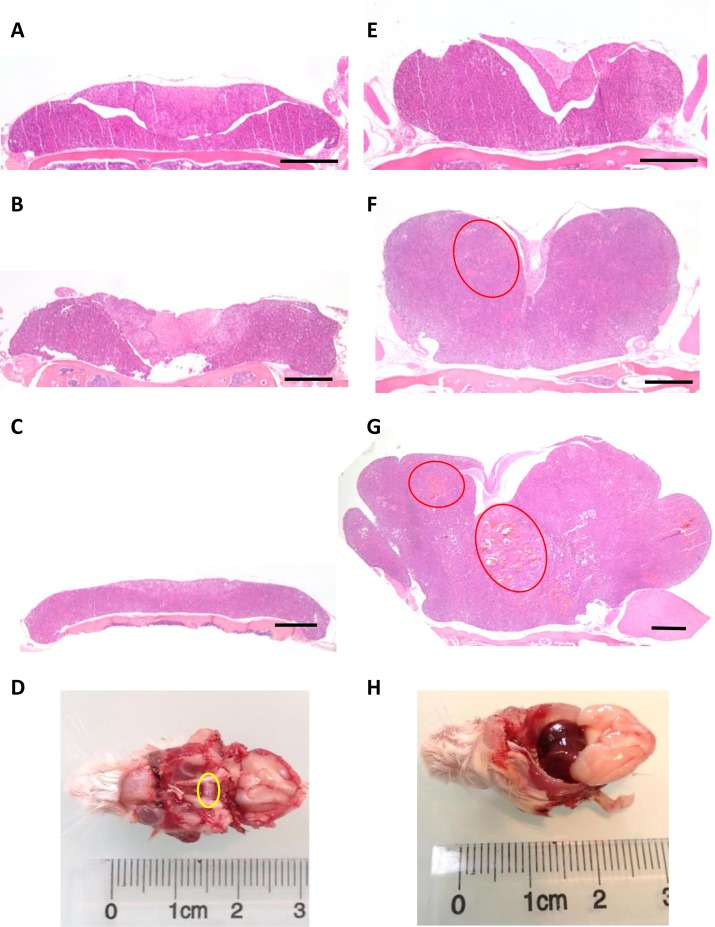
Pituitary enlargement in *Prlr*^–/–^ compared to *Prlr*^+/+^ mice Representative histological sections from *Prlr*^+/+^ of 4 (**A**), 12 (**B**), 18-month-old (**C**) and *Prlr*^–/–^ female mice of 4 (**E**), 12 (**F**) and 18 month-old (**G**). Red ellipses indicate cystic spaces and hypervascularisation. Bar scale = 500 μm. Macroscopical view of pituitary (yellow circle) of 24 month-old *Prlr*^+/+^ (**D**) and *Prlr*^–/–^ (**H**) female mice.

**Figure 4:**
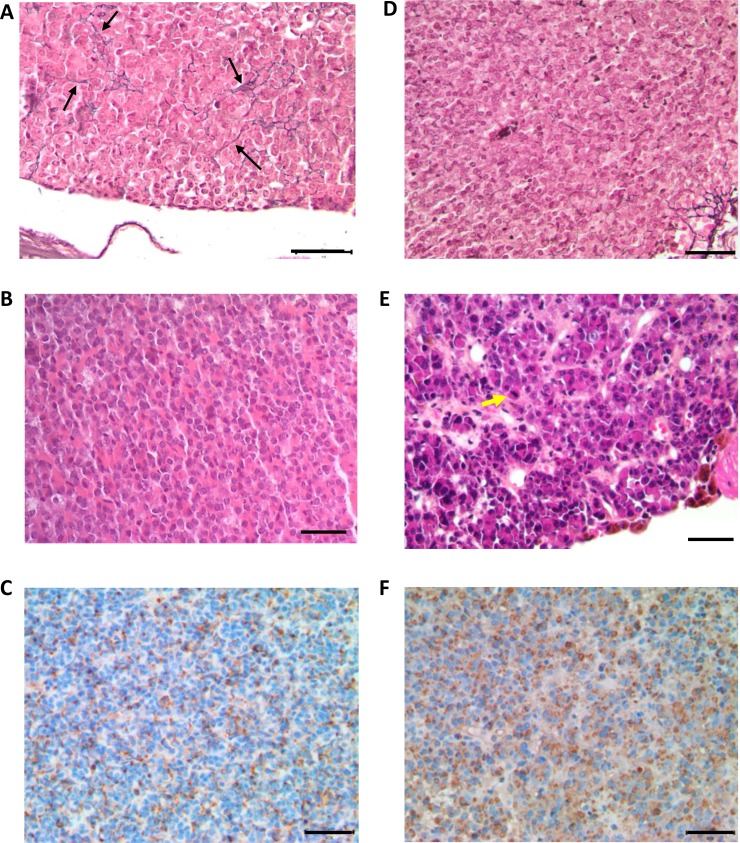
Histological analysis of anterior pituitaries from 24 month-old *Prlr*^+/+^ and *Prlr*^–/–^ mice Top row shows Gordon–Sweet silver staining in pituitaries sections of *Prlr*^+/+^ (**A**) and *Prlr*^–/–^ (**D**) mice. Black arrows indicate reticulin fibers. Middle row shows hematoxylin and eosin-stained pituitary sections of *Prlr*^+/+^ (**B**) and *Prlr*^–/–^ (**E**) mice. The wild-type pituitary has a normal architecture and mixture of cells (B). The *Prlr*^–/–^ genotype exhibits peliosis (extravasated erythrocytes not contained in capillaries) and lactotrophs with very large Golgi regions and scattered, large, hyperchromatic, atypical nuclei. Yellow arrow indicates mitosis (E). Bottom row: PRL immunohistochemistry. The wild-type pituitary (**C**) contains a few mature active lactotroph cells with PRL immunostaining. Almost all the cells in the *Prlr*^–/–^ pituitary (**F**) are active lactotrophs with PRL immunoreactivity. Bar scale = 50 µm.

In order to perform pituitary histological analysis, mice were sacrificed at 4, 12, 18 and 24 months (*n =* 6 in each group for both genotypes). At all ages, the development of both anterior and posterior pituitary was strictly normal in control (*Prlr*^+/+^) mice, with a well-visible intermediate lobe (Figure 3A–3D). In these control mice, the architecture was roughly acinar and reticulin staining showed a relatively developed peri-acinar fiber network, not identical to what is normally seen in adult human anterior pituitary (Figure 4A). The anterior pituitary cells were quite regular in shape and size without atypias (Figure 4B). The cytoplasm of most cells was acidophilic but some cells displayed basophilic cytoplasms. The nuclei were spherical with a little or absent nucleolus. Mitotic figures were absent (Figure 4B). PRL expression, assessed by immunohistochemistry, showed a normal expression with a “dot-like” pattern in lactotroph cells representing around 20–30% of total cells (Figure 4C). No evidence of pituitary adenoma was observed in any of the 25 *Prlr*^+/+^ studied mice.

The comparison between *Prlr*^+/+^ and *Prlr*^–/–^ mice revealed considerable differences in the size and shape of pituitary glands as well as in cytological aspects. Specifically, in 4-month-old *Prlr*^–/–^ the lateral wings of the anterior pituitary mice were already enlarged when compared to those of *Prlr*^+/+^ mice (Figure 3A and 3E). At this stage, cells were monomorphous, acidophilic, with some prominent nucleoli but no mitotic figures or evident adenoma were apparent. At ages of 12, 18 and 24 months, the pituitary glands of *Prlr*^–/–^ mice were drastically larger than those of *Prlr*^+/+^ mice (Figure 3F, 3G and 3H), with 100% tumor occurrence (*n =* 18). For instance, in 12-month-old *Prlr*^–/–^ mice, the lateral wings of the anterior pituitary were clearly enlarged with a solid and confluent growth pattern. Some cystic spaces were visible (Figure 3F). Anterior pituitary cells were monotonous, strongly acidophilic with voluminous nuclei and prominent nucleoli. These findings were suggestive of important hormone synthesis and secretion, related to acidophilic adenoma, and are in agreement with the very increased serum PRL levels. No evidence of normal residual anterior pituitary was seen. At 18 months of age, the lateral wings were even more and dramatically enlarged, inducing a compression of the adjacent structures such as the intermediate lobe, the posterior pituitary and the olfactory lobes. At this stage, histological examination showed that the anterior lobe was completely composed by a strongly acidophilic solid adenoma. Cystic spaces were frequent and hypervascularisation was observed (Figure 3G). At 24 months of age, adenomas further increased and measured approximately 7 mm of diameter, with hemorrhagic and necrotic aspect (Figure 3H). No evidence of bone invasion was found in any *Prlr*^–/–^ mice.

Mitotic activity was estimated to 2–3 mitosis 10 high-power fields (HPF) and 5–8 mitosis/10HPF in 18-month-old and 24-month-old respectively demonstrating an active process of cellular proliferation. Reticular network was completely absent (Figure 4D). Cellular atypias were seen and cells showed pleomorphic nuclei with evident and voluminous nucleoli (Figure 4E). An immunohistochemical analysis demonstrated a diffuse and intense PRL expression with a mainly “dot-like” Golgi pattern (Figure 4F).

Altogether, these biological, morphological and histological parameters illustrate that the absence of PRL receptor induces the development of prolactinoma from 12 months of age in females, with a penetrance of 100%. Thanks to pituitary MRI, a very attractive noninvasive imaging technology, anatomical tumor analysis was obtained. The present study constitutes the first characterization of such mouse pituitary adenomas by this technique. Among different subtypes of prolactinomas, *Prlr*^–/–^ pituitary adenoma mimics a human aggressive densely granulated macroprolactinoma, which is a highly secreting subtype. Indeed, adenomatous cells are actively secreting in this human tumor subtype much like in our model, as shown by PRL measurements and also immunohistochemical analysis. Moreover, the aggressiveness of *Prlr*^–/–^ prolactinomas is epitomized by their high mitotic activity. In addition and interestingly, this study showed that cabergoline potently suppresses hyperprolactinemia but did not induce tumor shrinkage after 3 months of treatment, suggesting that *Prlr*^–/–^ mouse could be an interesting model to improve our understanding of dopamine resistance in humans.

### Transcriptomics reveals common molecular alterations in *Prlr*^*–/–*^ mice and in estrogen-induced rat prolactinoma

To gain molecular insights into the pathogenesis of prolactinoma induced by the absence of PRLR, we performed a microarray analysis at 2 and 4 months of age (*n =* 12 for both genotypes), before the overt appearance of the tumors. Significance Analysis of Microarrays [17] showed that the age of the animals did not modify the gene expression patterns. Thus, we considered the transcriptomes of 2 and 4 months (*Prlr*^*+/+*^ or *Prlr*^–*/–*^) together to increase the statistical power of our analysis. This analysis detected 839 genes whose expression was modified by the *Prlr* knock-out, with a median false discovery rate <0.01, and undergoing an expression fold-change of at least 1.8. We obtained a list of 588 PRLR-activated targets (i.e., with a decreased expression in the absence of PRLR) and 251 repressed genes.

Next, we sought to identify the common set of genes dysregulated in our model and in another murine model of prolactinoma, to increase the robustness and expand the signification of our findings. For this, we focused on the model of 9 week-old male ACI rats treated with implants containing 5 mg of diethylstilbestrol (DES), which develop prolactinomas after 12 weeks of treatment. In these animals, pituitaries enlarge up to 10-fold and PRL levels increase up to 220 fold [18]. Specifically, we explored previously reported microarray data for this rat prolactinoma model (accession number GSE4028) [19] using the GEO2R program implemented in GEO (Gene Expression Omnibus). We focused on differentially expressed (DE) genes having adjusted *p*-values lower than 0.05. This analysis allowed us to obtain a list of 5647 DE genes (transcripts) in the pituitary of DES-treated ACI rats compared with vehicle treatment, 2576 of which were upregulated in the prolactinomas and 3071 down-regulated. The intersection of the rat and mouse datasets included an important number of common genes (303, which represent 36% of the mouse dysregulated genes). Then, we focused our attention only on the 218 genes displaying concordant variations (either up- or down-regulated in both models, Supplementary Table 1). A gene set enrichment analysis showed that the concordantly upregulated genes were characterized by keywords such as *cell cycle*, *regulation of cell proliferation*, *mitosis* (i.e., *Top2A, Rrm2, Cenpi, Bub1, Ska1, Spc25*). This result is consistent with an increased cell proliferation in *Prlr*^–*/–*^ pituitaries, which manifests anatomically as enlarged lateral pituitary wings at 4 months. Other enriched terms were *dopaminergic synapse* (i.e., *Creb3l1, Itpr3, Drd4, Gnai1*) and *estrogen signaling* (i.e., *Creb3l1, Itpr3, Gnai1*). This can be explained by the murine models themselves, since one of them is induced by an estrogen agonist, and the other is induced by disruption of PRL signaling, which is mostly controlled by dopamine. Regarding the concordantly down-regulated genes, the classification system showed that they were enriched in genes encoding *cell adhesion and related molecules* (i.e. *Cldn11, Alcam, Cdh2, Ncam1, Ncam2, Lrrc4c, Lrrc4b*). These findings are consistent with the fact that tumor expansion and invasion rely on increased cell proliferation and on changes in cell-to-cell adhesion. Indeed, several markers of cell adhesion have already been investigated and found dysregulated in prolactinomas [15, 20, 21].

### Potentially dysregulated downstream effectors in murine prolactinomas

**Figure 5:**
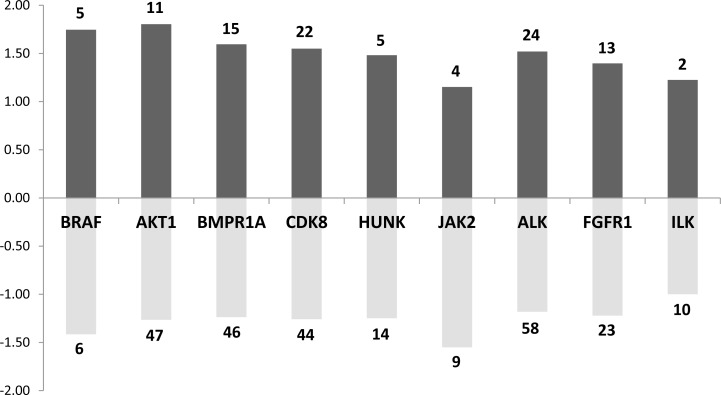
Main kinases whose known targets are dysregulated in both rat and mouse models Dark grey bar charts indicate for each kinase the mean fold change of up-regulated target genes. Pale grey bar charts indicate for each kinase the mean fold change of down-regulated target genes. The number of up or down-regulated target genes for each kinase are indicated in the bar charts.

To further explore which signaling pathways were perturbed in both mouse and rat prolactinoma models, we used the list of 218 concordantly dysregulated genes to search for enrichment in kinase targets. We focused our attention on kinases expressed in the pituitary (according to an arbitrary threshold set at 500 in the raw expression data). Many targets of JAK2 (13 targets) and AKT1 (58 targets) were found in the common DE genes (Figure 5). This is expected because both kinases participate in the canonical PRL signaling pathway [22]. Indeed, although the JAK/STAT pathway is considered as the major downstream pathways for PRLR signaling, PRL also activates the PI3K pathway, which involves AKT [23]. This view is supported by a recent pathway map depicting the PRLR-dependent signaling pathway generated from the analysis of 20 000 research articles [24]. In addition, the DE genes were enriched in targets of other 7 kinases (namely, BRAF, BMPR1A, CDK8, HUNK, ALK, FGFR1, ILK). This result suggests that either the *Prlr* deletion or DES treatment induces more complex signaling events than expected. For instance, the integrin-linked kinase (ILK) has emerged as a key transducer of β1-integrin signaling required for PRL induced differentiation of mammary epithelial cells, the target tissue of PRL [22]. ILK phosphorylates AKT1 and is thus required for signal transduction via PRLR [25]. These results taken altogether point to an activity of PRL signaling on lactotroph cells themselves.

Regarding the BRAF oncogene, it is known that it is commonly mutated in melanomas, papillary thyroid carcinomas and papillary craniopharyngioma [26], leading to constitutive activity in the Ras-mitogen-activated protein kinase (MAPK) pathway. Although no *BRAF* mutations have been found in pituitary adenomas, this kinase is overexpressed in non-functional pituitary adenomas, suggesting an over-activity of the Ras-B-Raf-MAPK pathway in these tumors [27]. Along similar lines, the tyrosine kinase Fibroblast Growth Factor Receptor 1 (FGFR1) was found to be highly expressed in pituitary tumors [28]. A significantly increased *Fgfr1* mRNA expression has been described in functioning tumors raising the possibility of using the FGFR1 as a molecular tumor marker.

Finally, BMPR1A is a receptor for BMP4, which plays a critical role in the formation of the anterior pituitary during embryonic development, as well as in the pathogenesis of adult pituitary tumors. In tumor cells, BMP4 promotes PRL secretion and lactotroph cell proliferation via a Smad-estrogen receptor (ER) crosstalk [29]. The modulation of BMP4 also plays an important role in the mechanism of action of dopaminergic agonists.

Interestingly, a enrichment analysis of these kinases shows that four of them (FGFR1, BRAF, JAK2 and AKT1) are linked to ERBB/EGFR pathway, and *ErbB4* expression level was found to be increased by approximately 2 times in *Prlr*^–/–^ mice compared to *Prlr*^+/+^ mice. This is consistent with the fact that ErbB/EGFR signaling is a determinant of PRL synthesis and lactotroph cell proliferation [30] and that its expression in prolactinomas is associated with tumor symptoms, invasion and response to dopamine agonists [31].

**Figure 6:**
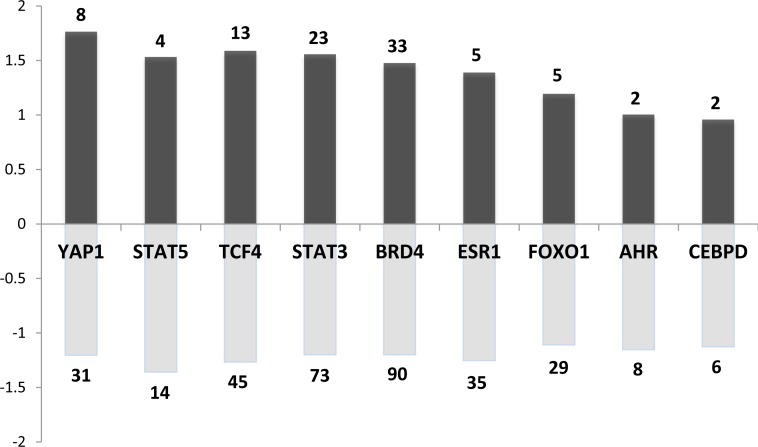
Main transcription factors (TF) whose known targets are dysregulated in both rat and mouse models Dark and pale grey bar charts indicate for each TF the mean fold change of up and down regulated target genes respectively. The number regarding the bar chart indicates the number of up or down-regulated target genes for each TF.

Regarding the potential downstream effectors of such kinases, we detected a series of 76 transcription factors (TFs) (expressed in the pituitary, i.e., >500 arbitrary units in our microarray data) whose known targets were dysregulated in both rat and mouse models. They are significantly involved in cancer and signaling pathways regulating pluripotency of stem cells (adjusted *p <* 10–4). A selection of the most interesting TFs is presented in Figure 6 along with the number of their potential targets and their averaged fold change in the mouse model.

Among these transcription factors, we found STAT5 (signal transducer and activator of transcription factors 5) and STAT3. This is consistent with the current view of PRL signaling being mediated by Jak2–STAT in the target tissues. This further supports the existence of an autocrine regulatory PRL loop in the pituitary.

Estrogen receptor alpha (ERα) was also retrieved by our analysis. ERα is an important physiological regulator of lactotroph cell proliferation. This has been demonstrated *in vivo* using ERα^–/–^ mouse model [32] and consistently, rat strains rapidly develop prolactinomas after estrogen exposure [18]. This underscores the importance of estradiol in the early stages of lactotroph tumorigenesis which would also be operating at 4 months in our mouse model.

Aryl hydrocarbon receptor (AhR) targets were also found to be dysregulated. AhR is known to mediate the effects of xenobiotics implicated in carcinogenesis [33]. In line with its potential implication in the murine model, it is the best-known interacting partner of the aryl hydrocarbon receptor-interacting protein (AIP), whose germline mutations predispose to pituitary somatotroph and lactotroph adenomas in human [34].

Known targets of the transcription factor CEBPD (CCAAT-enhancer-binding protein δ) also appeared dysregulated. Consistently, CEBPD has been previously identified as a critical gene regulating both PRL expression and lactotroph cell proliferation and is downregulated in prolactinomas [35]. Accordingly, *Cebpd* expression level was also decreased by approximately 5 times in our *Prlr*^–/–^ mice compared to *Prlr*^+/+^ mice. CEBPD regulates Cyclin D1 (CCND1), which was also detected as DE gene in our study (increased by 2.5 times in *Prlr*^–/–^) and dysregulated CCND1 is known to play a role in human pituitary tumorigenesis [36, 37]. It is worth noting that CCND1 potential targets were also DE in both mouse and rat models (*p <* 0.05). This is consistent with the fact that CCND1 plays a crucial role in the regulation of cell cycle and displays oncogenic properties.

Yes-associated protein (YAP) and the transcriptional coactivator with PDZ-binding motif (TAZ) belong to the Hippo signaling pathway and are phosphorylated by the LATS1 kinase. This cascade regulates the growth of tissues during development and plays a role in cancer. Indeed, activation of Hippo signaling results in dysplastic growth and increased organ size. It has been shown that *Lats1*^–/–^ mice exhibit a pituitary hyperplasia [38]. Thus, a dysregulation of the Hippo pathway may also contribute to pituitary tumorigenesis such as prolactinoma development.

FOXO1 (forkhead box transcription factor 1) plays a role in normal somatotrope differentiation as well as gonadotrope function. However, no role of this transcription factor has yet been described in lactotroph cell or in pituitary tumorigenesis [39]. That being said, it is worth mentioning that FOXO TFs are well-known targets of AKT1, and important regulators of cell-cycle and proliferation in many cell types.

We have also explored the intersection between the rat + mouse dataset and DE genes from microarray data of human prolactinomas obtained from a previous study [40]. The intersection included 12 genes (*Cebpd, Cdh2, Ghrhr, Gnrhr, Lgi1, Lrrn2, Plch2, Ppl, Stmn2, Tmem30b, Igsf1, Spc25*). Interestingly, among these 12 genes, 11 are targets of BRD4 which belongs to the BET family of nuclear proteins carrying bromodomains that are implicated in chromatin interactions. BRD proteins, and most prominently BRD4, are important regulators of MYCN transcription. A very recent study showed that the overexpression of MYCN induced pituitary tumors in mouse resembling human pituitary adenomas. Furthermore, treatment by JQ1, a BRD4-inhibitor, resulted in a reduction of tumor growth *in vivo,* suggesting that pituitary gland tumorigenesis is dependent on MYCN expression [41].

**Figure 7:**
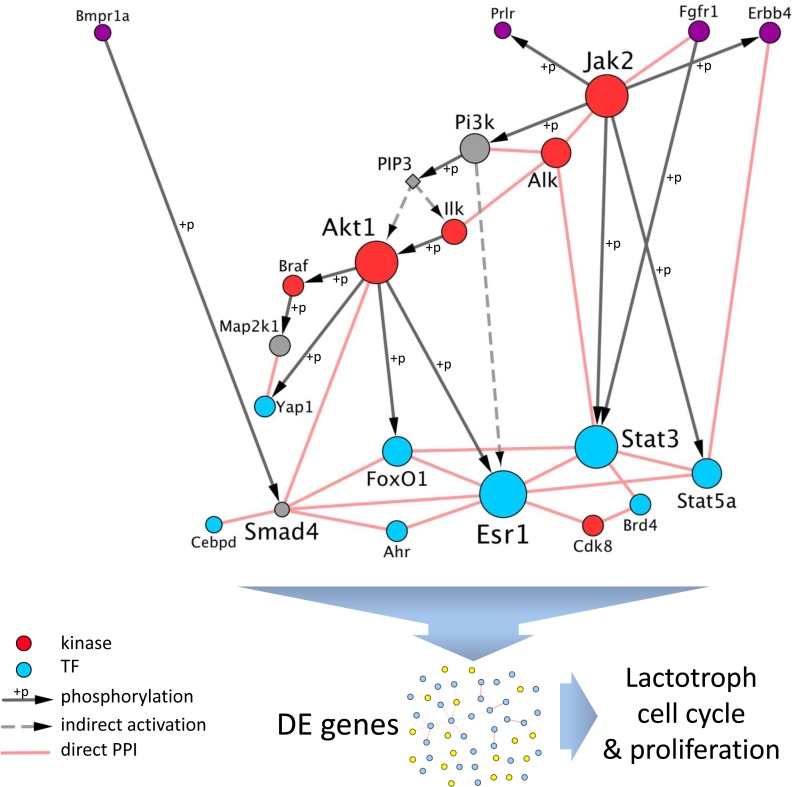
Interplay between regulators of DE genes downstream of PRLR The set of dysregulated genes both in *Prlr*^–/–^ mice and in DES-treated rats were significantly enriched in targets of kinases (in red) and FTs (in blue). Those regulators are connected by known direct protein-protein interactions (PPI) or are phosphorylation targets of another one (grey directed arrows). The network is rearranged with receptors at the top (in purple, note that BMPR1A and FGFR1 are also kinases), kinases as intermediate regulators, and FTs as downstream effectors. When PRLR function is abolished, the interplay between the regulators converges towards a dysregulation of their targets, as detected by transcriptomics, that results in altered cell cycle and proliferation of lactotroph cells.

In order to gain insights into the interplay between those various regulators (namely, kinases and TFs), we built a network, by retrieving known direct protein-protein interactions (i.e., interactome) among them using Cytoscape. Interestingly, when using this list of 18 kinases and TFs as input, 16 appeared directly connected to at least another regulator (Figure 7). Moreover, several of the TFs are known to be phosphorylated by at least one of the kinases. This network highlights the strong interplay between the potential effectors of PRLR, both at the kinases and FTs levels, to coordinate the transcriptomic response observed in the two rodent models.

The molecular events involved in the pathogenesis of prolactinomas are still a matter of debate. Here, we demonstrate the key role of PRLR signaling in initiating prolactinoma, since *Prlr*^–/–^ mice develop lactotroph adenomas with 100% penetrance from 12 months of age. Although its role in the mammary gland has previously been established [42, 43], our study provides evidence that a perturbation of PRLR signaling triggers pituitary adenoma development. Combined biological and histological analysis of *Prlr*^–/–^ mouse adenomas constitute an interesting approach to better understand the human disease despite their phenotypic differences. *Prlr*^–/–^ prolactinomas constitute a model of aggressive disease because of their high mitotic activity. The absence of tumor shrinkage after 3 months of cabergoline administration suggests that *Prlr*^–/–^ prolactinomas could be a model of the dopamine-resistant subtype. Additionally, molecular analyses performed at an early stage, before tumor occurrence, enabled us to identify a molecular network in pathways that emerge from the absence of PRLR and result in lactotroph adenoma several months later. This strategy results in finding candidate genes that could be involved in PRL pituitary tumors initiation. The *Prlr*^–*/–*^ mouse model will help identify the multiple steps involved in pituitary tumorigenesis and test novel therapeutic approaches such as tyrosine kinase inhibitors that are already effective in the targeted treatment of various tumors. Mouse pituitary MRI, as suggested by our study, will open new avenue for tumor response evaluation to such novel therapeutic options.

## MATERIALS AND METHODS

### Hormonal analysis in mouse

Blood was collected from the tail vein of mice, immediately diluted in PBS-T (PBS, 0.05% Tween20), and then promptly frozen and stored until use at −20°C. PRL concentrations were measured using a home-made ultrasensitive-ELISA, as previously reported [44]. The animal facility was granted approval (N°C94-043-12), given by the French Administration (Ministère de l′Agriculture). All procedures were approved by the local ethic committee Consortium des Animaleries Paris Sud (CAPSud) (N°2012-021).

### Pituitary MRI scanning

Gas anesthesia was induced using isoflurane delivered in air at a flow of 4 L/min (Isovet, Centravet Plancoët, France) and then with maintenance concentration at 1 to 0.5 L/min and magnetic resonance imaging (MRI) was performed with a. 4.7 Tesla (T) Bruker system (Biospec 47/40 USR Bruker, Ettlingen, Germany). A dose of 125 µL Dotarem® (0.5 mM/mL gadolinium) was administered intraperitoneally and T1 fat sat imaging acquisition was performed 10 minutes later. Coronal sections of 300µm were analyzed using Radiant Dicom viewer.

### Cabergoline treatment

For drug response, blood was collected from two 12-month-old female *Prlr*^–/–^ mice. Then, 1 mg/kg/day cabergoline was administered orally during three months. Post-treatment blood sample and pituitary MRI scanning were obtained after one month and three months of treatment.

### Pathological analysis

In order to evaluate initial and delayed histological abnormalities along prolactinoma development, mice were sacrificed at ages 4, 6, 12, 18 and 24 months, and their pituitary glands as well as the sella were inspected visually with a dissecting microscope and placed immediately in 10% zinc formalin for 24 hours. After rapid decalcification (pituitary glands and sella were placed in 50 mL DC3 histological decalcifier from VWR chemicals during 10 minutes), paraffin-embedded serial sections 4–5 μm thick were cut with a standardized protocol and stained with hematoxylin and eosin or the Gordon–Sweet silver method for reticulin matrix. Immunohistochemical staining to identify lactotroph cells was performed using the streptavidin-biotin peroxidase technique. Primary antiserum directed against PRL was used at the 1:20 000 dilution (provided by A.F. Parlow and the National Hormone and Peptide Program) further diluted 1:20 (DAKO Corp., Carpinteria, California, USA).

### Transcriptome analysis

Two and four month-old wild-type (*n =* 6 and 6 respectively) and *Prlr*^–*/–*^ (*n =* 6 and 6 respectively) mice were sacrified by decapitation. Pituitaries were removed, pooled three by three to form duplicates for each genotype, and frozen at –80°C before RNA extraction. Fifty ng total RNA was amplified and labeled with Cy3 using Low input Quick Amp labeling Kit (Agilent technologies, Santa Clara, California, USA) according to manufacturer’s instructions. Labeled cRNA was then fragmented and hybridized over 1 Sureprint G3 Mm9 8 × 60 K array (Agilent technologies), allowing the study of the 8 samples in parallel and interrogating over 40K annotated transcripts of Mm9 assembly. Hybridization was performed according to manufacturer’s instructions. Raw fluorescence data were extracted using Genespring software v12 (Agilent technologies) and normalized using the 75th percentile method. Values were then log2 normalized and subtracted of the median value for each replicate. The whole experiment was replicated with another set of animals (12 wild-type and 12 *Prlr*^–/–^ mice). Differential gene expression (DE) was assessed using Significance Analysis of Microarrays [17]. The DE gene list was explored using the gene set enrichment analysis program Enrichr [45] to identify upstream regulators (kinases and transcription factors) for which known targets are significantly enriched in DE genes. This analysis was performed on 25 March 2016. The network was constructed using Cytoscape 3.3 and the GeneMania plugin to automatically retrieve known direct protein-protein interactions (interactome) from databases and manually curated bibliographic sources [46, 47] and additional manual search.

## SUPPLEMENTARY MATERIALS TABLE




