# Supplementary material for: Natural and molecular history of prolactinoma: insights from a Prlr –/– mouse model
Source: Oncotarget. 2017 Dec 27;9(5):6144–55. doi: 10.18632/oncotarget.23713 (PMC5814201; doi:10.18632/oncotarget.23713)
Supplement: Supplementary file 1 [file oncotarget-09-6144-s001.pdf]

Natural and molecular history of prolactinoma: insights from a *Prlr*$^{-/-}$ mouse model

SUPPLEMENTARY MATERIALS

Supplementary Table 1: List of 218 genes displaying concordant variations in mouse at rat prolactinoma models. See Supplementary_Table_1
